# Single Phase Dual-energy CT Angiography: One-stop-shop Tool for Evaluating Aneurysmal Subarachnoid Hemorrhage

**DOI:** 10.1038/srep26704

**Published:** 2016-05-25

**Authors:** Qian Qian Ni, Chun Xiang Tang, Yan E Zhao, Chang Sheng Zhou, Guo Zhong Chen, Guang Ming Lu, Long Jiang Zhang

**Affiliations:** 1 Department of Medical Imaging, Jinling Hospital, Medical School of Nanjing University, Nanjing, Jiangsu, 210002, China

## Abstract

Aneurysmal subarachnoid hemorrhages have extremely high case fatality in clinic. Early and rapid identifications of ruptured intracranial aneurysms seem to be especially important. Here we evaluate clinical value of single phase contrast-enhanced dual-energy CT angiograph (DE-CTA) as a one-stop-shop tool in detecting aneurysmal subarachnoid hemorrhage. One hundred and five patients who underwent true non-enhanced CT (TNCT), contrast-enhanced DE-CTA and digital subtraction angiography (DSA) were included. Image quality and detectability of intracranial hemorrhage were evaluated and compared between virtual non-enhanced CT (VNCT) images reconstructed from DE-CTA and TNCT. There was no statistical difference in image quality (*P* > 0.05) between VNCT and TNCT. The agreement of VNCT and TNCT in detecting intracranial hemorrhage reached 98.1% on a per-patient basis. With DSA as reference standard, sensitivity and specificity on a per-patient were 98.3% and 97.9% for DE-CTA in intracranial aneurysm detection. Effective dose of DE-CTA was reduced by 75.0% compared to conventional digital subtraction CTA. Thus, single phase contrast-enhanced DE-CTA is optimal reliable one-stop-shop tool for detecting intracranial hemorrhage with VNCT and intracranial aneurysms with DE-CTA with substantial radiation dose reduction compared with conventional digital subtraction CTA.

In today’s clinical practice, MR imaging seems to represent the cornerstone of neuroimaging because of its tissue characterization in the brain and functional imaging such as perfusion weighted imaging, diffusion weighted imaging, and MR spectroscopy. However, since it is relatively time-consuming and costly, CT remains preferred for the acutely ill patients presenting with intracranial hemorrhage or patients with contraindications to MR imaging^1^,^2^,^3^.

Dual-energy CT is the product of ongoing development of CT technology^4^. It has been increasingly and widely accepted by clinicians due to its additional attenuation measurement and various post-processing opportunities^1^,^2^,^5^,^6^. These techniques used in dual-energy CT were reported to increase image quality, improve diagnostic accuracy and reduce the radiation exposure^6^,^7^,^8^. Automated bone removal technology is one of the most appealing applications of dual-energy CT, which allows direct visualization of iodinated vessels without increase of radiation exposure^9^,^10^. In addition to bone removal, removing the iodine component allows for the creation of virtual non-enhanced CT (VNCT) image^11^,^12^,^13^. In this approach, the conventional true non-enhanced CT (TNCT) examinations might be able to be omitted. Other clinically relevant applications such as blood pool imaging^14^, urinary stone characterization^15^ and virtual monoenergetic imaging^16^ have also been used in many clinical trials to optimize the patients’ management.

Approximately 1–5% adults are in the danger of intracranial aneurysms in the United States. Rupture of an intracranial aneurysm causes 85% subarachnoid hemorrhages^17^. Such hemorrhages have high case fatality and morbidity, especially for relatively young patients less than 65 years old^18^. Compared to conventional angiography, CT angiography (CTA) is highly efficient for identifying intracranial aneurysms and planning surgical therapies^19^, DE-CTA showed potentially superior diagnostic accuracy for evaluation of intracranial aneurysms to conventional CTA^10^,^20^. Furthermore, dual-energy CT can be beneficial for iodine component removal to reconstruct VNCT images, which has been shown to replace the conventional TNCT and reduce radiation exposure^12^,^16^. Although both techniques have been reported to separately detect intracranial aneurysm and subarachnoid hemorrhages, to the best of our knowledge, no study has been performed using single phase DE-CTA combining VNCT with bone removal techniques to optimize CT workflow for screening patients with aneurysmal subarachnoid hemorrhage. This is very implicated for the critically ill patients.

Hence, the purpose of this study is to evaluate clinical value of single phase DE-CTA in detecting aneurysmal subarachnoid hemorrhage, which combines VNCT to evaluate hemorrhage focus and bone removal CTA technique to detect intracranial aneurysms with TNCT and DSA as reference standard, respectively.

## Results

### Image quality

Mean attenuation, noise, SNR, and CNR values of TNCT and VNCT images are presented in Table 1. The mean attenuation of bleeding focus was 61 ± 10 HU for TNCT, and 56 ± 11 HU for VNCT. The mean SNR and CNR of TNCT were 19 ± 6 and 10 ± 5, and 16 ± 4 and 7 ± 3 of VNCT. The mean attenuation, SNR and CNR of VNCT were lower than those of TNCT (all *P* < 0.01).

Subjective image scores of two series of non-enhanced images and DE-CTA by two independent readers are presented in Table 2. Kappa coefficients for inter-reader reliability between the two independent readers ranged from 0.474 to 0.824, interpreted as moderate (0.41–0.60) to excellent (0.81–1.00) agreement. Of non-enhanced CT, the image quality rated excellent (score = 4) was awarded in 95 patients (90.5%) of VNCT, and in 100 patients (95.2%) of TNCT; the image rated good (score = 3) was awarded in 10 patients (9.5%) of VNCT, and in 5 patients (4.8%) of TNCT. There was no significant difference for subjective overall image quality evaluation between TNCT and VNCT (*P* = 0.166). Of DE-CTA, the image quality rated excellent (score = 4) was awarded in 88 patients (83.8%), good (15.2%, 16/105), and poor (1.0%, 1/105).

### Diagnostic performance

#### Hemorrhage detection

Among the 105 patients, 58 patients had 101 bleeding focus based on TNCT. There were 11 patients with subarachnoid hemorrhage, 32 patients with subarachnoid hemorrhage combined with other bleeding focus (i.e., intracerebral hematoma, ventricular hematoma and others), 15 patients with other intracranial hematoma. Of 101 bleeding focus, 43 were subarachnoid hemorrhage, 17 lesions were intracerebral hematoma, 32 lesions were ventricular hematoma, and 9 were other lesions such as epidural hematoma.

**Figure 1:**
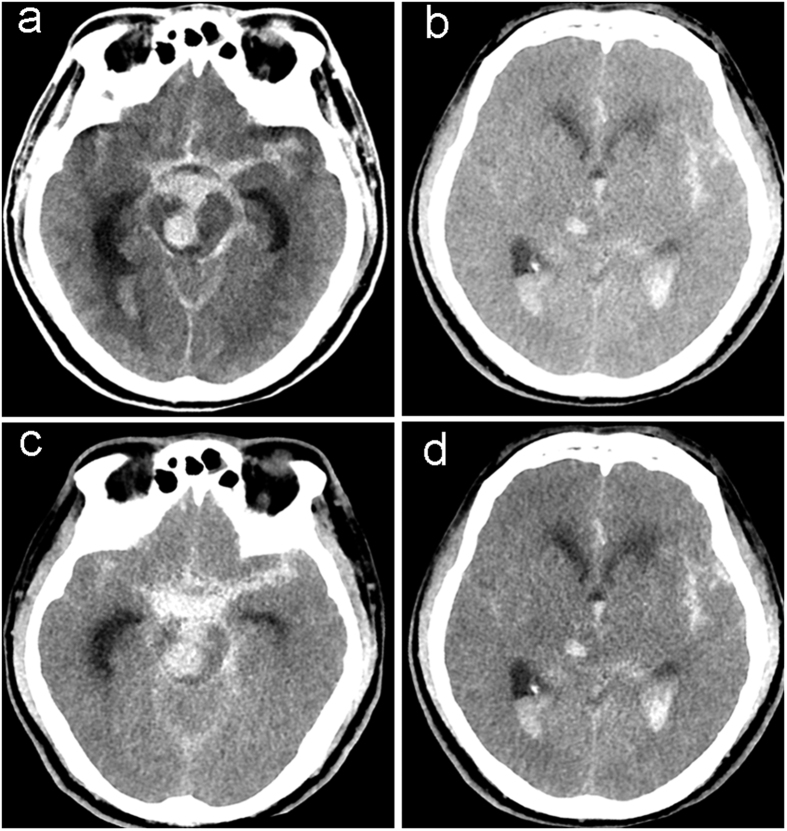
A 59-year-old man with spontaneous subarachnoid hemorrhage and ventricular hematoma caused by ruptured aneurysm of right anterior superior cerebellar artery. (**a,b**) True non-enhanced CT image, (**c,d**) virtual non-enhanced CT image.

**Figure 2:**
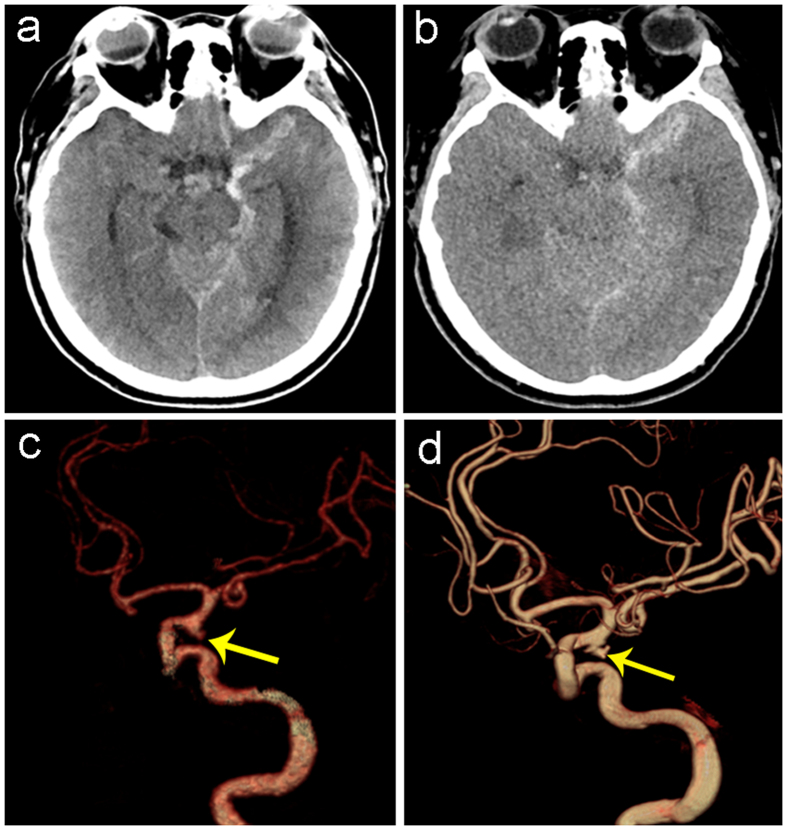
A 59-year-old women with spontaneous subarachnoid hemorrhage caused by ruptured aneurysm in left posterior communicating artery. (**a**) True non-enhanced CT image and (**b**) virtual non-enhanced CT image show subarachnoid hemorrhage, (**c**) volume-rendered dual-energy CTA image shows a true-positive aneurysm in the left posterior communicating artery (yellow arrow), which was confirmed by 3D-DSA (**d**).

VNCT correctly detected 98 bleeding lesions in 57 patients against TNCT reference standard. The sensitivities and specificities for detecting hemorrhage on a per-patient basis were 98.3% and 97.9%, and 97.0% and 95.8% respectively on a per-lesion basis (Table 3, Figs 1 and 2). There were no statistically differences in diagnostic accuracy between virtual and true non-enhanced CT on a per-patient and per-lesion basis (both *P* > 0.99). Sensitivities grouped by bleeding focus were 97.8%, 98.4%, 97.4%, and 100%, and specificities were 95.8%, 100%, 100%, and 100%, respectively (Table 3). There was no difference in diagnostic accuracy for detecting different bleeding lesions between virtual and true non-enhanced CT (all *P* > 0.50).

#### Aneurysms detection

**Figure 3:**
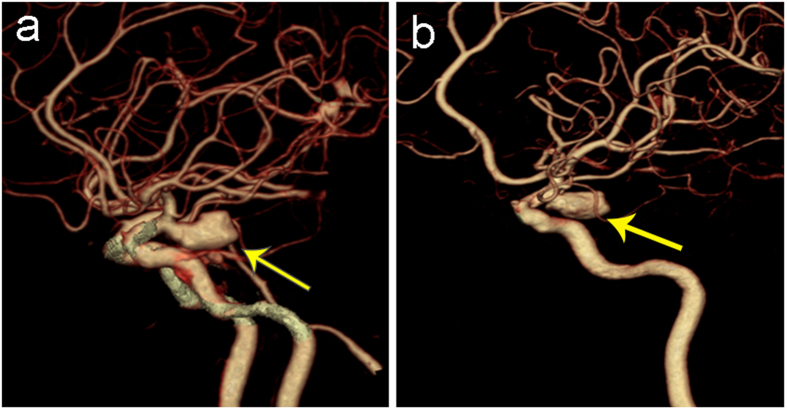
A 55-year-old women with a giant aneurysm in left posterior communicating artery. (**a**) Volume-rendered dual-energy CTA image shows a true-positive aneurysm (arrow) in the posterior communicating artery, which was confirmed by 3D-DSA (**b**).

There were 69 aneurysms in 58 patients evaluated by DSA as reference standard. DE-CTA correctly detected 65 aneurysms in 57 patients. Table 4 reports that the sensitivity and specificity for detecting aneurysms by DE-CTA were 98.3% and 97.9% on a per-patient basis, while 97.1% and 95.8% on a per-aneurysm basis. It did not show any statistical difference in diagnostic accuracy of DE-CTA and DSA in assessment of aneurysms whether on a per-patient or per-aneurysm basis (Figs 2 and 3).

Of the 69 aneurysms detected by DSA, 24 were smaller than 3 mm, 34 were 3–8 mm, and 11 were larger than 8 mm. Moreover, 43 aneurysms of them were located in anterior circulation, and 26 aneurysms were located in posterior circulation. The missed 2 aneurysms by DE-CTA were both smaller than 2 mm in anterior and posterior circulation, respectively, while the misdiagnosed 2 aneurysms were smaller than 3 mm and larger than 8 mm, respectively (Table 4). The McNemar test did not show any significant differences of sensitivity and specificity in detecting aneurysms of different sizes and locations between DE-CTA and DSA (all *P* > 0.99). Furthermore, DE-CTA in our study showed a high sensitivity and specificity (91.7% and 97.9%, respectively) for small aneurysm detection (Supplementary Fig. S1).

### Radiation dose

Table 5 reports the mean CTDIvol, DLP, and ED of TNCT, dual-energy CT, and the sum of values of TNCT and dual-energy CT that was taken as the radiation dose of conventional digital subtraction CTA. The mean CTDIvol, DLP, and ED of single phase contrast-enhanced dual-energy CT scan were 14.6 ± 1.4 mGy, 238.5 ± 29.9  mGy*cm, and 0.5 ± 0.1 mSv, respectively. Nevertheless, when coupled with a non-enhanced CT examination, the mean CTDIvol, DLP, and ED changed to 59.8 ± 2.4 mGy, 965.4 ± 67.5, mGy*cm, and 2.0 ± 0.1 mSv, respectively. So compared with conventional digital subtraction CTA, the CTDIvol, DLP, and ED was reduced by approximately 75.6%, 75.3% and 75.0%, respectively.

## Discussion

The results of our study showed that, at significant radiation dose reducing and time saving, the new imaging protocol demonstrated in our study by combining VNCT with DE-CTA is comparable to the TNCT and DSA in terms of image quality and diagnostic accuracy of aneurysmal subarachnoid hemorrhage.

Conventional digital subtraction CTA has been widely accepted as the primary examination choice for patients suspected of intracranial aneurysms^19^,^21^,^22^. However, the limitations of it still be questioned by the minority, such as additional radiation exposure by a non-enhanced CT examination, patient movements between the 2 scans, and incomplete bone removal at the skull base. DE-CTA appears to be a promising modality to mitigate the above-mentioned limitations of digital subtraction CTA. Watanabe *et al.*^20^ first introduced the bone removal application of DE-CTA for evaluating intracranial aneurysms in 12 patients. They stated that 3 of 9 aneurysms adjacent to the skull base were fully visible in DE-CTA but only partially visible in conventional CTA. Besides, the calcifications removal by dual-energy CT allowed a precise analysis of luminal structure of aneurysms than DSA. Zhang *et al.*^23^,^24^ published 2 articles about the diagnostic accuracy of intracranial aneurysm detection of DE-CTA in comparison with digital subtraction CTA and 3D DSA in 46 and 80 patients, respectively. They reported no statistical difference of image quality between DE-CTA and digital subtraction CTA. The sensitivity and specificity of aneurysm detection were 95.0–95.7% and 95–100% on a per-aneurysm basis with DSA as reference standard. DE-CTA reduced the radiation dose by approximately 60% compared to digital subtraction CTA.

The results of our study were similar to the previous studies in aneurysms detection by DE-CTA^10^,^23^,^24^. Our study demonstrated high sensitivity and specificity for intracranial aneurysms detection by DE-CTA when compared with DSA in a relatively large patient population. Furthermore, our study first gave an accurate diagnostic accuracy in smaller aneurysm (<3 mm) detection by DE-CTA, as the smaller aneurysms involved in the previous studies were all less than 10 cases^20^,^23^,^24^. For neuroradiologists, smaller intracranial aneurysm has always presented particular technical challenges. The diagnostic accuracy of smaller intracranial aneurysms detection was controversial for either MR angiography or CT angiography^19^,^25^,^26^,^27^. In our study, the 2 false-negative aneurysms were all smaller than 2 mm in diameter. The sensitivity and specificity for detecting smaller aneurysms were 91.7% and 97.9%, respectively. This result demonstrated that the diagnostic accuracy of smaller aneurysm detection by DE-CTA was comparable to digital subtraction CTA, which was based on a large patient size^19^,^28^. Besides, aneurysms located in vessels adjacent to skull base were easier to be missed by conventional CTA due to its limited bone removal technique. DE-CTA was reported superior to conventional CTA in detecting aneurysm adjacent to skull base^20^. This was convinced in our study as DE-CTA detected all the skull base aneurysms against DSA reference standard. One missed small aneurysm (<2 mm) in our study was located in anterior superior cerebellar artery. This reminded us that small aneurysms in uncommon locations were extremely easy to be missed.

The reconstruction of VNCT image is based on material quantification by dual-energy CT, which removes the iodine component from enhanced CT images^29^. This application is desirable in the CT evaluation of various clinical settings such as kidney, liver, and pulmonary diseases^11^,^13^,^30^. Conventional non-enhanced CT and MR imaging both have limitations in hemorrhage detection, as they can not differentiate hemorrhage from contrast medium. It makes VNCT a promising application in intracranial hemorrhage detection^12^,^31^. Ferda *et al.*^12^ first evaluated the VNCT technique in screening the intracranial hemorrhage. Their results stated that the image quality was found to be sufficient in 96% (12/13) VNCT images, and the intracranial bleeding detection accuracy was 96% in per-lesion basis and 100% in per-patient basis. However, the patient size of their study was limited, and no negative cases might cause an overestimation of the sensitivity. A recent study^32^ evaluated the ability of VNCT in diagnosing subarachnoid hemorrhage in 84 patients. Comparisons between VNCT and TNCT in this study showed no difference in subarachnoid hemorrhage detection at both “individual level” (i.e., based on the CT scan as whole for a given patient) and “lesion level” (i.e., on the basis of 4 different bleeding regions on the CT scan). The CTDIvol was reported to reduce significantly when TNCT was omitted. There were still 3 patients with subarachnoid hemorrhage missed by VNCT, which were attributed to small size of bleeding sites, low number of hemorrhage focus, higher image noise, and long examination interval. Brisman^33^ stated that despite high sensitivity and specificity of VNCT, the missed diagnosis of even one aneurysm could lead to a significant possibility of neurologic devastation, which might outweigh any potential benefit from radiation spared. Furthermore, he stated that etiology diagnosis was secondarily to hemorrhage diagnosis, because the management of hemorrhage was less controversial. Thus, obtaining CTA first upended the workflow.

In the clinical setting, the etiology of subarachnoid hemorrhage will be further investigated with CTA or DSA if non-enhanced CT demonstrated fatal subarachnoid hemorrhage. It was the etiology of vascular lesions that determined the treatment planning of subarachnoid hemorrhage and preventative methods of rebleeding. For example, microaneurysm in the posterior inferior cerebellar artery was difficult to detect because of the tortuous arteries and various branches. Its diagnosis relied on the consistency of suspected lesions and bleeding focus. When there were no signs of subarachnoid hemorrhage, CTA was the most important modality to work-up a potential aneurysm in posterior inferior cerebellar artery. Hence, DE-CTA, as a one-stop-shop tool, should be the first-choice technique to screen subarachnoid hemorrhage together with aneurysms. The results of our study demonstrated that the high sensitivity, specificity and image quality of VNCT image made it a promising tool to replace TNCT, although there did exist some limitations of VNCT images such as the lower CT attenuation of hematomas and the suboptimal image delineation of small lesions, which affected the diagnosis of small hemorrhage locus. In our study, 2 of the missed bleeding focus were located in ventricle, combining with subarachnoid bleeding. However, the aneurysms of the 2 patients were all detected by DE-CTA, which were confirmed by DSA and surgery. Besides, there was no hemorrhage focus in TNCT of the 2 false negative cases of aneurysms detection, in which the aneurysms were smaller than 2 mm in diameter. Our results demonstrated that DE-CTA could be the first choice to evaluate suspected aneurysmal subarachnoid hemorrhage by simultaneously providing VNCT and DE-CTA images in a single phase contrast-enhanced CTA in dual-energy mode scanning.

Our study had some limitations. First, we did not compare the image quality and diagnostic accuracy of intracranial aneurysms detection between DE-CTA and conventional digital subtraction CTA. The previous investigators^23^ obtained the conventional CTA images by average weighted 120 kVp images reconstructed from DE-CTA and post-processing opportunities for digital subtraction CTA images. However, we think it was not ideal for this comparison between subtraction CTA from the average weighted 120 kVp images and non-enhanced CT images and DE-CTA. On the other hand, we thought it was unethical to persuade the patients to undergo additional CTA scanning, while some bias might be introduced if we set a conventional CTA group as reference. Second, the true radiation doses of conventional digital subtraction CTA examinations were not reported in our study because we did not set a conventional CTA group. In our study, we added up the CTDIvol of TNCT and contrast-enhanced dual-energy CT scan and took the sum as CTDIvol of conventional CTA. Our result of radiation dose reduction was consistent with that of the previous study^24^. However, the actual radiation dose value of dual-energy CT and conventional enhanced CT was not in full agreement. Third, the patient size of our study limited the generalization of our results. In our study, the aneurysmal subarachnoid hemorrhage accounted for a large percentage of hemorrhage, while hemorrhage caused by other cerebral vascular events such as vascular malformations were too little. So we could not conclude whether the high diagnostic accuracy could be kept if this new imaging protocol by combining VNCT with DE-CTA was applied in other cerebral vascular abnormalities. So a larger patient size is probably warranted to verify more applications of this new imaging protocol. Last, VNCT images visually showed a little higher image noise compared with TNCT and this might affect the diagnosis of microhemorrhage. However, there was no significant difference in detecting SAH between the two protocols in our study.

In conclusion, at significant radiation dose reducing and time saving, our study suggests a useful contribution of single phase contrast-enhanced DE-CTA for both the detection of hemorrhage with virtual non-enhanced CT and the detection of intracranial aneurysms with DE-CTA in the emergent setting. This is very implicated in critically ill patients with suspected aneurysmal subarachnoid hemorrhage because of simplified CT workflow, high diagnostic accuracy for detecting both intracranial hemorrhage and aneurysms, and substantial reduction of radiation dose.

## Materials and Methods

### Patients

Approved by the Medical Research Ethics Committee of Jinling Hospital, 105 consecutive patients (46 male and 59 female; mean age, 50 ± 13 [SD] years) were included in our study between April 2013 and August 2015. Written informed consent was obtained from all patients or their legal guardian. Patients were enrolled in this study if they were clinically suspected subarachnoid hemorrhage or aneurysms, i.e., patients presented with severe headache, vomiting, or a lowered level of consciousness, or suspicion of intracranial aneurysm after medical examinations. Our study was carried out in accordance with the relevant guidelines. All patients should undergo both TNCT and DE-CTA within 3 days before DSA (conventional and/or 3D-DSA). Exclusion criteria for this study were history of prior reaction to iodinated contrast media, hemodynamic instability, renal insufficiency (i.e., creatinine level > 120 mol/L), and under the age of 18.

### CT imaging

#### CT acquisition and image reconstruction

All CT examinations were performed in a second-generation dual-source CT scanner (Somatom Flash; Siemens Healthcare, Forchheim, Germany). First, a conventional non-enhanced CT covering the lower jaw to the vertex of the head was acquired using a routine automatic tube current modulation (Care Dose 4D) with the following parameters: tube potential 120 kVp, effective tube current 300 mA, pitch 0.6, rotation time 0.5 second, collimation 64 × 2 × 0.6 mm, and reconstruction slice thickness width 5 mm with a reconstruction increment of 5 mm. Then, CT angiography was performed in the dual-energy mode using 140 kVp tube voltage and 112 effective milliampere second for measurement system A and 80 kVp tube voltage and 224 effective milliampere second for measurement system B, respectively; 0.33-second rotation time; 32 × 2 × 0.6 mm collimation; and a pitch of 0.7. For vessel enhancement, 60 mL iodinated contrast medium (iopromide, Ultravist, 300 mg I/mL, Bayer Schering Pharma, Berlin, Germany) with subsequent 40 mL of saline solution were injected into the antecubital vein at a flow of 4.0 mL/s. The scanning was begun 4 second after CT threshold value reached to 100 HU for triggering. The region of interest was placed in the internal carotid artery.

The 140 and 80 kVp images were reconstructed separately with a slice thickness of 0.75 mm at 0.5 mm increments using a Q30f kernel for a field of view of 180 mm. Sinogram affirmed iterative reconstruction (SAFIRE, Siemens) at strength level 3 was employed in all cases. The dual-energy images (140 and 80 kVp images) were loaded onto a workstation (Multi Modality Workplace; Siemens Medical Solutions, Erlangen, Germany). By using the brain hemorrhage virtual non-enhanced (VNC) application in default setting, iodine was subtracted from the enhanced CT image, resulting in a VNCT image. Consistent with TNCT images, the VNCT images were reconstructed with 5 mm section thickness and 5 mm increment in the same window and level settings. Meanwhile, by using “head bone removal” application, bone removal CTA images were obtained. The dedicated software of Inspace (Multi Modality Workplace; Siemens Medical Solutions, Erlangen, Germany) was then used to reformat the three-dimensional images by volume rendering (VR), multiplanar reconstruction (MPR), and maximum intensity projection (MIP).

The volume CT dose index (CTDIvol, mGy) and dose-length product (DLP, mGy*cm) were recorded from the existing patient protocol. The effective radiation dose (ED, unit in mSv) was derived by multiplying DLP with the weighting value (κ), a conversion factor for head CT imaging (κ = 0.0021 mSv/mGy*cm).

#### CT image quality evaluation

Both VNCT and TNCT images were transferred to a dedicated workstation (Multi Modality Work Siemens). One radiologist (Q.Q.N. with 3 years experience in neuroradiology) performed all non-enhanced CT measurements independently. The CT attenuation values of hemorrhagic focus were measured using a user-defined circular region of interest (ROI) with an area of 0.1–0.2 cm^2^ in the largest slice of the lesion for each case. The radiologist prescribed three independent ROIs to mitigate partial volume effects and operator dependent measurements. ROIs of 1 cm^2^ were placed in the white matter to measure the attenuation values of the brain parenchyma, which was selected as background. Meanwhile, the standard deviation (SD) was selected as image noise. Signal-to-noise ratio (SNR) was calculated as SNR_a_ = CTnumber_lesion_/SD, while contrast-to-noise ratio (CNR) was calculated as CNR_a_ = (CTnumber_lesion_ − CTnumber_parenchyma_)/SD^34^.

All subjective image quality evaluation of non-enhanced CT and DE-CTA was performed in consensus by two radiologists (L.J.Z and C.X.T. with 15 and 8 years’ experience for interpretation in neuroradiology studies, respectively). The overall image quality of non-enhanced CT and DE-CTA was rated according to a 4-point scale (1 = poor; 2 = moderate; 3 = good, 4 = excellent), which in turn contained two parts respectively^12^,^23^,^35^. For non-enhanced CT (TNCT and VNCT), image graininess (1 = marked unacceptable noise level; 2 = average image noise; 3 = below average noise; 4 = absent perceivable noise) and image delineation (1 = hardly visible lesion; 2 = subtle but detectable lesion; 3 = easily detectable; 4 = excellent delineation or structures contours) were evaluated. For DE-CTA, quality of bone removal (1 = large bone remnants; 2 = partial bone remnants; 3 = only tiny bone remnants; 4 = no bone remnants evaluation of vasculatures) and depiction of vascular structures (1 = blurring of vascular structures; 2 = moderate delineation of vascular structures; 3 = more than moderate delineation of vascular structures; 4 = perfect delineation of vascular structures) were evaluated.

#### Hemorrhage and intracranial aneurysm evaluation

The same two neuroradiologists performing the subjective image analysis independently evaluated the presence or absence of intracranial hemorrhage and aneurysms on two series of non-enhanced CT images and DE-CTA. In case of disagreement, another two experienced neuroradiologists (Y.E.Z. and C.S.Z. both with 15 years of experience) were invited to reach the final diagnosis.

The presence of subarachnoid hemorrhage, intracerebral hematoma, ventricular hematoma and other hemorrhage focus such as epidural hematoma on both TNCT and VNCT images were recorded. The readers first evaluated the TNCT images followed by VNCT after 2 months.

Aneurysms were measured according to the diameters (<3 mm, 3–8 mm, and >8 mm) and recorded by locations (anterior circulation: anterior cerebral arteries, middle cerebral arteries, internal carotid arteries, and anterior choroidal arteries; posterior circulation: vertebral and basilar arteries, posterior communicating arteries, posterior cerebral arteries, anterior superior cerebellar arteries, and posterior inferior cerebellar arteries).

### DSA imaging

DSA was performed in all 105 patients involved using a biplane DSA unit with rotational capabilities by femoral catheterization (AXIOM Artis dTA; Siemens Medical Systems, Forchheim, Germany). Before removing the catheter in target vessel(s) with confirmed or suspected aneurysm(s), a single 3D-DSA acquisition was obtained. Then the angiographic data were transferred to an adjacent 3D workstation (Siemens) for reconstruction of 3D-DSA images. All the angiographies, aneurysms detection, and aneurysms measurement were performed by a group of highly experienced interventional neuroradiologists (non-authors, with more than 10 years of neuroangiographic experience).

## Statistical analysis

Statistical evaluation was performed using SPSS software (SPSS, version 16, SPSS Inc., Chicago, IL, USA). Continuous variables were expressed as mean ± SD and categorical variables were expressed as frequencies or percentages. Paired t-test was used to compare mean attenuation, noise, SNR and CNR of TNCT and VNCT images. Kappa analysis was used to evaluate the inter-reader agreement for assessing the image quality. Strength of consistency based on the κ value was interpreted as follows: <0.2, poor; 0.21–0.40, fair; 0.61–0.80, good; 0.81–1.00, excellent. The Wilcoxon test was used to compare the scores of qualitative grading between the two series non-enhanced CT images. With TNCT and DSA used as reference standard for detection of intracranial hemorrhage and aneurysms, respectively, the sensitivity, specificity, positive predictive value (PPV), negative predictive value (NPV), accuracy and the corresponding 95% confidence interval (CI) were calculated on a per-patient, per-lesion, and per-aneurysm basis. The ability of VNCT and DE-CTA to detect intracranial hemorrhage focus and aneurysms were compared using the McNemar test. *P* < 0.05 was considered to indicate a statistically significant difference.

## Additional Information

**How to cite this article**: Ni, Q. Q. *et al.* Single Phase Dual-energy CT Angiography: One-stop-shop Tool for Evaluating Aneurysmal Subarachnoid Hemorrhage. *Sci. Rep.* **6**, 26704; doi: 10.1038/srep26704 (2016).

## Supplementary Material

Supplementary Information

## Figures and Tables

**Table 1:** Objective image quality measurement in two series of non-enhanced images.

Protocols	TNCT	VNCT	*P* value
CT value (HU)	61 ± 10	56 ± 11	<0.01
Noise (HU)	4 ± 2	4 ± 1	0.564
SNR	19 ± 6	16 ± 4	<0.01
CNR	10 ± 5	7 ± 3	<0.01

Data are presented as mean ± SD.

TNCT = true non-enhanced CT; VNCT = virtual non-enhanced CT.

**Table 2:** Subjective image quality scores of non-enhanced CT and DE-CTA by 2 independent readers.

Image Dataset	Reader 1	Reader2	Kappa Coefficient (k)
TNCT			
Image graininess	3.95 ± 0.21	3.93 ± 0.25	0.669
Image delineation	3.96 ± 0.19	3.97 ± 0.17	0.694
Overall image quality	3.95 ± 0.21	3.97 ± 0.17	0.502
VNCT			
Image graininess	3.91 ± 0.28	3.90 ± 0.31	0.824
Image delineation	3.92 ± 0.27	3.97±0.17	0.557
Overall image quality	3.90 ± 0.30	3.95 ± 0.21	0.481
DE-CTA			
Bone removal	3.57 ± 0.57	3.50 ± 0.56	0.474
Depiction of vascular structures	3.80 ± 0.51	3.86 ± 0.43	0.815
Overall image quality	3.82 ± 0.48	3.81 ± 0.46	0.689

Data are presented as mean ± SD.

TNCT = true non-enhanced CT; VNCT = virtual non-enhanced CT; DE-CTA = dual-energy CT angiography.

**Table 3:** Intracranial bleeding detection with virtual non-enhanced CT compared to a true non-enhanced CT as reference standard.

Approach	Results (n)				Statistical Analysis (%)				
	TP	TN	FP	FN	Sensitivity	Specificity	PPV	NPV	Accuracy
Per-patient	57	46	1	1	98.3 (90.9, 99.7)	97.9 (88.9, 99.6)	98.3 (90.9, 99.7)	97.9 (88.9, 99.6)	98.1 (93.3, 99.5)
Per-lesion	98	46	2	3	97.0 (91.6, 99.0)	95.8 (86.0, 98.9)	98.0 (93.0, 99.5)	93.9 (83.5, 97.9)	96.6 (92.4, 98.6)
Subarachnoid hemorrhage	43	46	2	0	100 (91.8, 100)	95.8 (86.0, 98.9)	95.6 (85.2, 98.8)	100 (92.3, 100)	97.8 (92.3, 99.4)
Intracerebral hematoma	16	46	0	1	94.1 (73.0, 99.0)	100 (92.3, 100)	100 (80.6, 100)	97.9 (88.9, 99.6)	98.4 (91.5, 99.7)
Ventricular hematoma	30	46	0	2	93.8 (79.9, 98.3)	100 (92.3, 100)	100 (88.7, 100)	95.8 (86.0, 98.9)	97.4 (91.1, 99.3)
Others	9	46	0	0	100 (70.1, 100)	100 (92.3, 100)	100 (70.1, 100)	100 (92.3, 100)	100 (93.5, 100)

Data are presented as mean ± SD.

TP = true positive; TN = true negative; FP = false positive; FN = false negative; PPV = positive predictive value; NPV = negative predictive value.

**Table 4:** Aneurysm detection with DE-CTA with DSA as reference standard.

Approach	Results (n)				Statistical Analysis (%)				
	TP	TN	FP	FN	Sensitivity	Specificity	PPV	NPV	Accuracy
Per-patient	57	46	1	1	98.3 (90.9, 99.7)	97.9 (88.9, 99.6)	98.3 (90.9, 99.7)	97.9 (88.9, 99.6)	98.1 (93.3, 99.5)
Per-aneurysm	67	46	2	2	97.1 (90.0, 99.2)	95.8 (86.0, 98.9)	97.1 (90.0, 99.2)	95.8 (86.0, 98.9)	96.6 (91.5, 98.7)
<3 mm	22	46	1	2	91.7 (74.2, 97.7)	97.9 (88.9, 99.6)	95.7 (79.0, 99.2)	95.8 (86.0, 98.9)	95.8 (88.3, 98.6)
3–8 mm	34	46	0	0	100 (89.9, 100)	100 (92.3, 100)	100 (89.9, 100)	100 (92.3, 100)	100 (95.4, 100)
>8 mm	11	46	1	0	100 (74.1, 100)	97.9 (88.9, 99.6)	91.7 (64.6, 98.5)	100 (92.3, 100)	98.3 (90.9, 99.7)
Anterior circulation	42	46	0	1	97.7 (87.9, 99.6)	100 (92.3, 100)	100 (91.6, 100)	97.9 (88.9, 99.6)	98.9 (93.9, 99.8)
Posterior circulation	25	46	2	1	96.2 (81.1, 99.3)	95.8 (86.0, 98.9)	92.6 (76.6, 97.9)	97.9 (88.9, 99.6)	95.9 88.8, 98.6)

Data are presented as mean ± SD.

TP = true positive; TN = true negative; FP = false positive; FN = false negative; PPV = positive predictive value; NPV = negative predictive value.

**Table 5:** Radiation dose comparison between the two different CT protocols.

Protocols	TNCT	DE-CTA	TNCT+DE-CTA	*P* value
CTDIvol (mGy)	45.2 ± 2.0	14.6 ± 1.4	59.8 ± 2.4	<0.01
DLP (mGy*cm)	726.9 ± 45.0	238.5 ± 29.9	965.4 ± 67.5	<0.01
ED (mSv)	1.5 ± 0.1	0.5 ± 0.1	2.0 ± 0.1	<0.01

Data are presented as mean ± SD.

TNCT = true non-enhanced CT: DECT = dual-energy CT; CTDIvol = Volume CT dose index; DLP = dose-length product; ED = Effective dose.
