# Supplementary material for: Single Phase Dual-energy CT Angiography: One-stop-shop Tool for Evaluating Aneurysmal Subarachnoid Hemorrhage
Source: Sci Rep. 2016 May 25;6:26704. doi: 10.1038/srep26704 (PMC4879615; doi:10.1038/srep26704)
Supplement: Supplementary Information [file srep26704-s1.pdf]

Single Phase Dual-energy CT Angiography: One-stop-shop Tool for Evaluating Aneurysmal Subarachnoid Hemorrhage

Qian Qian Ni[1], Chun Xiang Tang[1], Yan E Zhao[1],

Chang Sheng Zhou[1], Guo Zhong Chen[1], Guang Ming Lu[1], Long Jiang Zhang*[1]

[1] Department of Medical Imaging, Jinling Hospital, Medical School of Nanjing University, Nanjing, Jiangsu, 210002, China

Corresponding authors

Long Jiang Zhang, Department of Medical Imaging, Jinling Hospital, Medical School of Nanjing University, Nanjing, Jiangsu, 210002, E-mail: kevinzhlj@163.com Tel#: 86-25-80860185. Fax#: 86-25-84804659

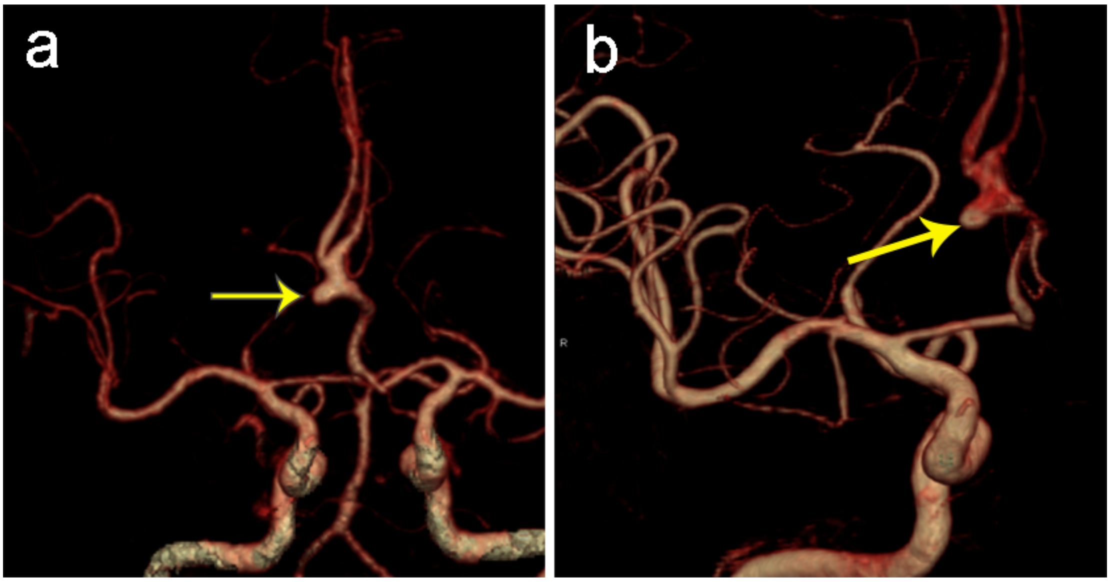

Supplementary Fig. S1 A 49-year-old women with a small aneurysm in right anterior cerebral artery. (a) Volume-rendered dual-energy CTA image shows a true-positive aneurysm (arrow) in the right anterior cerebral artery, which was confirmed by 3D-DSA (b).
